# Editorial: World Schizophrenia awareness day: raising awareness and advancing pharmacological strategies in Schizophrenia

**DOI:** 10.3389/fphar.2026.1820307

**Published:** 2026-03-10

**Authors:** Ciria C. Hernandez, Valéria de Almeida, Michael J. Marino

**Affiliations:** 1 Feinberg School of Medicine, Northwestern University, Chicago, IL, United States; 2 University Hospital Muenster, Department of Psychiatry, Muenster, Germany; 3 Neuroscience, Merck & Co, Inc., West Point, PA, United States

**Keywords:** antipsychotic adherence, antipsychotic-induced metabolic effects, early intervention, integrated care model, personalized psychiatry, Schizophrenia, systems-based care, treatment resistance

## 1 Editorial perspective: the evolution of Schizophrenia management

The landscape of schizophrenia research is undergoing a fundamental strategic shift. While traditional models have long centered on dopaminergic dysfunction, contemporary research is moving toward a more integrated paradigm that encompasses metabolic dysfunction, the nuances of treatment resistance, and the highly individualized nature of the patient journey. This evolution reflects growing recognition that dopamine-targeting pharmacotherapies, while effective for positive symptoms, often fail to address the debilitating negative and cognitive symptoms that drive long-term disability and highly correlate with quality-of-life measures, including job loss, homelessness, and suicide. Crucially, the “individualized patient journey” is no longer viewed merely as a qualitative metric but as a vital clinical predictor of treatment success and functional trajectory.

In observance of World Schizophrenia Awareness Day, the psychiatric community is challenged to move beyond the antiquated “stabilize and discharge” model of care. This editorial synthesizes six pivotal studies to raise awareness and advance pharmacological strategies, framing a fundamental shift in the clinical paradigm. The field is undergoing an encouraging shift from reactive symptom suppression to a proactive, more effective approach that prioritizes metabolic health, long-term adherence, and early identification of treatment resistance. This Research Topic underscores that successful long-term outcomes are not merely a product of biochemical efficacy but are rooted in the strategic integration of patient experience and provider foresight.

## 2 Stakeholder perceptions and the LAI patient journey

The ADVANCE Study (Franzenburg et al.) provides a qualitative roadmap for navigating the complexities of Long-Acting Injectable (LAI) adoption. The study identifies two distinct “Patient Profiles” that determine the long-term success of treatment. Profile 1 involves early symptom management within a robust outpatient support structure, leading to higher LAI acceptance. Conversely, Profile 2 is defined by severe acute episodes and traumatic hospitalizations. Strategically, the location of treatment initiation is the primary determinant of adherence; inpatient initiation, often associated with “trauma from prior forced injection,” creates a profound barrier to future LAI uptake. Furthermore, a critical strategic gap exists in education: 89% of caregivers were unaware of LAIs prior to clinician introduction, despite the fact that these treatments prioritize quality-of-life over mere symptom management.

## 3 Neurometabolic frontiers: ketogenic therapy as a strategic adjunct

Ketogenic Therapy (KT) is a significant differentiator in treating the “negative and cognitive symptoms” that remain largely untouched by traditional D2-targeting agents. The rationale for KT is to bypass the “prefrontal cortical hypofunction” and the impairments in glucose metabolism inherent in the schizophrenic brain (Chaves et al.). Because the brain consumes 20%–25% of the body’s resting glucose, synaptic instability is an inevitable consequence of impaired glucose utilization. KT shifts the primary energy substrate to ketone bodies—primarily β-hydroxybutyrate (BHB)—to restore mitochondrial energy flux. Strategically, KT serves a dual purpose: it targets the biological roots of cognitive decline while potentially mitigating the metabolic syndrome often induced by the very drugs used to treat the disorder. However, the efficacy of any metabolic intervention is often limited by patient compliance and adherence to dietary changes, as well as the clinical reality of long-term treatment resistance.

## 4 Mapping the knowledge domain of treatment-resistant Schizophrenia

Bibliometric analysis of the Treatment-Resistant Schizophrenia (TRS) domain indicates that 30% of patients fail to respond to initial therapy, and another 10%–60% develop resistance over time (Cai et al.). A strategic shift toward Early Recognition of TRS is a prognostic imperative. Data indicate that delayed initiation of clozapine (≥20 years post-diagnosis) significantly increases the risk of rehospitalization. Therefore, delaying evidence-based treatment for TRS is not a conservative clinical choice; it is a prognostic failure that exacerbates the disease’s functional burden. Effective TRS management is often complicated by secondary physiological burdens, most notably endocrine disruptions, which require constant vigilance.

## 5 Managing the endocrine burden: hyperprolactinemia risk stratification

Hyperprolactinemia (HPRL) is an underrecognized but prevalent endocrine condition, affecting 63.99% of hospitalized schizophrenia patients (Yang et al.). Chronic HPRL is a major clinical risk factor for osteoporosis, cardiovascular disease, and gynecological tumors. Strategic management requires moving beyond reactive care toward a proactive risk-stratification model. The severity of HPRL is positively correlated with “prolactin-raising” agents such as Sulpiride, Amisulpride, and Risperidone. Conversely, the clinical imperative for individualized management favors “prolactin-sparing” strategies, specifically the use of Aripiprazole. As a D2 partial agonist, Aripiprazole restores tonic inhibitory control over prolactin secretion in the anterior pituitary. Individualized endocrine management is a cornerstone of high-quality care, mitigating the risk that pharmacological treatment may impose a physical burden as disabling as the psychiatric symptoms themselves.

## 6 Measuring what matters: the Antipsychotics Functional Index

The Antipsychotics Functional Index (AFI) was developed to comprehensively assess the functional impact of antipsychotic treatment in schizophrenia, addressing the limitations of traditional symptom-centered measures (Marinescu et al.). The study found that AFI effectively captures essential aspects of daily functioning—such as cognitive performance, social engagement, and occupational capacity—beyond what standard symptom rating scales convey. AFI scores demonstrated strong validity, correlating with established quality-of-life measures and showing partial independence from symptom severity. Importantly, AFI distinguished between antipsychotic agents based on their functional outcomes. Longitudinal analyses revealed that AFI is sensitive to treatment changes over time and may better predict real-world recovery than symptom-based metrics. Overall, the findings support the AFI as a valuable tool for evaluating antipsychotic effectiveness and guiding personalized treatment in schizophrenia.

## 7 Metabolic and redox neuroprotection in antibiotic therapy

Antibiotic-associated neurotoxicity leads to neurologic and psychiatric adverse effects, particularly affecting elderly, critically ill, and renal-impaired patients. This review highlights that mechanisms such as oxidative stress, mitochondrial dysfunction, neuroinflammation, and excitatory–inhibitory imbalances contribute to central nervous system toxicity caused by common antibiotics, including β-lactams and fluoroquinolones (Lounici). The authors suggest two protective strategies: N-acetylcysteine (NAC) to restore glutathione and improve redox balance, and ketone-based metabolic support to enhance mitochondrial function and neurotransmission. These approaches aim to address the vulnerabilities that predispose high-risk patients to neurotoxicity. Further clinical studies are needed to validate risk assessment and neuroprotective strategies during high-risk antibiotic therapy.

## 8 Synthesis: toward a complete strategic framework

The current way we care for people with schizophrenia is evolving to become more effective by focusing on three main areas: making sure treatment is delivered reliably, helping restore metabolic health, and closely monitoring physical wellbeing. Recent studies have brought these areas into focus:

1. Reliable Treatment: This involves using injections that last a long time to ensure patients receive their medication consistently.
2. Metabolic Health: Many people with schizophrenia can face challenges related to weight and other health issues, so improving their overall metabolic health is important.
3. Physical Monitoring: Keeping an eye on hormone levels and other physical markers helps to catch any potential issues early on.

As we move forward, it is clear that managing schizophrenia will require a more personalized approach that pays attention to each individual’s needs. This comprehensive strategy aims to help people achieve better long-term recovery and a higher quality-of-life.

